# Neurocardiology: Cardiovascular Changes and Specific Brain Region Infarcts

**DOI:** 10.1155/2017/5646348

**Published:** 2017-07-03

**Authors:** Rongjun Zou, Wanting Shi, Jun Tao, Hongmu Li, Xifeng Lin, Songran Yang, Ping Hua

**Affiliations:** ^1^Department of Cardiovascular Surgery, Sun Yat-sen Memorial Hospital, Sun Yat-sen University, Guangzhou 510120, China; ^2^Department of Gastroenterology, The Fifth Affiliated Hospital, Sun Yat-sen University, Zhuhai 519000, China; ^3^The Biobank of Sun Yat-sen Memorial Hospital, Sun Yat-sen University, Guangzhou 510120, China; ^4^Department of Experimental Psychology, University of Oxford, Oxford OX1 3UD, UK; ^5^Guangdong Province Key Laboratory of Brain Function and Disease, Zhongshan School of Medicine, Sun Yat-sen University, Guangzhou 510080, China

## Abstract

There are complex and dynamic reflex control networks between the heart and the brain, including cardiac and intrathoracic ganglia, spinal cord, brainstem, and central nucleus. Recent literature based on animal model and clinical trials indicates a close link between cardiac function and nervous systems. It is noteworthy that the autonomic nervous-based therapeutics has shown great potential in the management of atrial fibrillation, ventricular arrhythmia, and myocardial remodeling. However, the potential mechanisms of postoperative brain injury and cardiovascular changes, particularly heart rate variability and the presence of arrhythmias, are not understood. In this chapter, we will describe mechanisms of brain damage undergoing cardiac surgery and focus on the interaction between cardiovascular changes and damage to specific brain regions.

## 1. Introduction

Effective cerebral protection remains the principle concern during cardiac surgery. In patients undergoing cardiac surgery, postoperative brain injury can contribute to increased morbidity and mortality and has negative effects on the quality of life and costs. With advances in medical and technological technologies, cardiac surgery can be performed on more complicated cases with several comorbidities. However, the risk of brain injury after surgery is also increased in these patients [1]. For example, heart disease patients show a high incidence of neurological symptoms (40–60%) including depression and/or depressive symptoms, epilepsy, and forgetfulness [2].

In fact, the primary cause of neurological injury in these patients is thought to relate to formation of an embolus due to gaseous, organic, or inorganic particles generated during the cardiac surgery which lodge in cerebral arteries [3, 4]. Due to the resulting acute cerebral ischemia, damage to the nucleus tractus solitarius (NTS) can negatively impact on the nucleus ambiguus, dorsal motor nucleus of the vagus, hypoglossal, parabrachial, and locus coeruleus, the hippocampus, entorhinal cortex, prefrontal cortex, amygdala, insula, and many key nuclei in the brainstem. Furthermore, the resulting neuronal and synaptic dysfunction in the NTS may affect its interconnected pathways, impacting on almost the entire central nervous system (CNS) [5]. Other types of structural brain injury, including scattered cerebral infarcts and specific regions of gray matter volume loss, can also occur following cardiac surgery and may contribute to the physiological and behavioral deficits in cardiovascular autonomic responses. For example, cortical autonomic dysfunction may indirectly influence cardiac function via altering centrally mediated autonomic output to the heart in patients with chronic epilepsy [6].

## 2. Mechanisms of Brain Injury with On-Pump Cardiac Surgery

The major causes involved in neurologic injury during cardiac surgery are microemboli, hypoperfusion, and inflammatory reaction, which can occur in the CPB time or any time of perioperative period for a variety of reasons. It has long been known that the brain is highly sensitive to hypoxia and, correspondingly, is threatened by thromboembolic ischemia. With regard to microemboli, the major types causing brain injury are air, fat, and vascular debris, which are distributed in blood flow and cause neuronal injury by blocking cerebral vessels and then leading a hypoperfusion during on-pump cardiac surgery [7]. Of note, cerebral microemboli cause larger lesions at higher temperatures, which may be associated with an acute inflammatory reaction.

In this scene, long CPB time initiates a cascade of inflammatory reactions due to the contact of patient blood with external surfaces of extracorporeal circulation machine or surgical stimulation. Additionally, circulating particles directly activate systemic inflammation via contacting with platelets, white blood cells, and endothelial cells. As a result, activated various proinflammatory cytokines, including TNF-alpha, IL-1, IL-6, IL-8, or prostaglandin, are released from platelets, leucocytes, and endothelial cell [8]. Furthermore, another interesting point to discuss is Toll-like receptors (TLRs) [9]. It is widely accepted that TLRs are an important mediator of autophagy and the neuroinflammatory cascade, which contribute to the loss of blood-brain-barrier and behavioral impairment [10, 11]. Endogenous ligands released from ischemic neurons can activate TLR signaling pathways, while release of systemic proinflammatory mediators can activate microglia, resulting in further release of inflammatory cytokines and secondary inflammatory injury [12]. By contrast, the preconditioning phenomena induced by a minor cerebral ischemic event or by TLR ligands can reduce the degree of TLR-mediated inflammation and brain injury following secondary, more severe cerebral ischemia [13].

Additionally, during hypoperfusion, hypoxia leads to anaerobic glycolysis and activation of proapoptotic pathways. There is also a concurrent accumulation of lactate, intracellular calcium overload, and release of reactive oxygen species (ROS), excitatory amino acids (EAA), and inflammatory cytokines in myocytes [14]. ROS are oxygen-derived compounds that serve as a marker for oxidative/nitrosative stress response in relation to brain damage after ischemia. Furthermore, accumulation of ROS could break cellular homeostasis and interfere with cytochrome oxidase, leading to destruction of mitochondria structure and impairment of mitochondrial respiration [7, 8]. On the other hand, ROS can be generated by arachidonic acid, nitric oxide, catecholamines, glutamate, and activation of N-methyl-d-aspartate receptors. These compounds lead to an influx of sodium and chloride into neurons, causing secondary intracellular secondary damage, and an influx of water into the cell, resulting in intracellular edema and neuronal death [1, 6, 7].

In the phase of reperfusion of cerebral lesions, the main energy substrates are dramatically altered; metabolites accumulation causes mitochondrial changes, such as altering the inner membrane potential that disturbs the distribution of ions, leading to intracellular calcium overload, and causing the collapse of mitochondrial membrane structures which opens the mitochondrial permeability transition pore (mPTP) [8]. On one hand, rapid transformation of mitochondria generates an enormous amount of highly reactive and destructive ROS that affect electron transference in the respiratory chain, which dissipates and uncouples the electrochemical gradient of the inner mitochondrial membrane. On the other hand, due to the failure of ATP-dependent ion pumps and intracellular calcium overload, mitochondrial apoptosis-inducing factors (AIF) and cytochrome C are released during open mPTP. Both molecules are involved in programmed mitochondrial damage and activate the caspase-cascade reactions, which in turn increase intracellular osmolarity and volume and activate apoptin and aggravate the damage of membranaceous structures and organelles [1, 7]. In summary, it can be stated that these changes can be accompanied by a series of pathways and regulatory molecular mechanisms, such as mitochondrial apoptotic pathways, autophagy, and necrosis.

In fact, in the on-pump cardiac surgery, different pharmacological and nonpharmacological methods to better preserve the brain are still in investigation and many strategies are available to reduce cerebral damage, including adequate anticoagulation, deep hypothermic temperature, or in-depth or screen filters in arterial inflow and venous outflow [7, 8].

## 3. Predictors of Brain Injury with On-Pump Cardiac Surgery

In order to identify better indicators of brain injury severity after heart surgery, Özatik et al. compared complement proteins, interleukins, white blood cells, and S100*β* protein before the initiation of CPB, immediately prior to aortic cross-clamping, following unclamping, and at postoperative 1st and 24th hours in 40-patient prospective cohort study. The authors found that high serum level of S100*β* protein is associated with increased levels of serum inflammatory mediators and systemic inflammatory response during CPB [15]. Additionally, Demir et al. analysed thirty patients' serum neuron-specific enolase (NSE), interleukin-6 (IL-6), and IL-10 before CPB and 4 and 24 hours after the end of extracorporeal circulation and found that serum cytokine and NSE levels were significantly associated with neuronal damage [16].

However, whether serum neuromarkers (NSE, S100*β*) have a predictive effect on the adverse neurological outcome has not been concluded yet. Sanchez-de-Toledo et al. measured cerebral regional oxygen saturation (rSO2) and serum biomarkers such as NSE, S100*β*, glial fibrillary acidic protein (GFAP), and brain-derived neurotrophic factor (BDNF) in 39 neurologically normal patients undergoing cardiac surgery with CPB. They prospectively observed adverse neurological outcomes via pediatric cerebral performance category (PCPC) score at 12 months after surgery and found that cerebral rSO2 was significantly associated with neurological impairment, while none of the serum neuromarkers (NSE, S100*β*, GFAP, and BDNF) in this cohort appear to be helpful [17]. Similarly, the explorative analysis from a randomized clinical trial, SafeBoosC II, showed that none of the S100*β*, brain-fatty-acid-binding-protein, and neuroketal were associated with the burden of either cerebral hypoxia or hyperoxia [18]. As described above, there is a debate on the effect of serum neuromarkers (NSE, S100*β*) in adverse neurological outcome with on-pump cardiac surgery.

## 4. Interaction between Cardiovascular Changes and Specific Brain Regions

In 1985, Natelson [19] described a new interdisciplinary area termed “neurocardiology,” which examines the interaction between the cardiovascular and autonomic nervous systems in pathological states. Modern neuroimaging data, including positron emission tomography and functional magnetic resonance imaging, show a complex set of neural interactions termed the neurocardiac axis, consisting of the insular cortex, the anterior cingulate cortex, the prefrontal cortex, and the amygdala, an area connected to other regions involved in autonomic control [20, 21]. Baroreflex sensitivity, dynamic electrocardiogram (ECG) changes, and heart rate variability (HRV) are also important parameters in understanding the influence of the autonomic system on both heart and brain activity [22].

### 4.1. Cardiovascular Changes and the Insular Cortex

As the insular cortex is located in the region of the middle cerebral arteries, it is more susceptible to injury following cerebrovascular disease. Indeed, ~33% of patients with hemispheric cerebral ischemia exhibited an insular infarct [23]. Damage to the insular cortex damage is associated with arrhythmia, disruption in diurnal blood pressure variation, myocardial injury, and breathing disorders during sleep [24], as well as elevated plasma levels of brain natriuretic peptide, catecholamines, and neuropeptide Y (NPY) [25, 26].

The insular cortex is a core region for interoceptive processing, including cardiac perception, emotion, and awareness, and for self-consciousness [27, 28]. The insular cortex also plays a crucial role in the central autonomic network and is typically associated with autonomic sympathetic activation [29]. In imaging studies examining cortical gray matter volumes, a smaller size of the bilateral insula predicted the magnitude of autonomic change following infarction [30]. The alterations in interoceptive or exteroceptive environmental cues observed in psychiatric disorders can also have serious cardiovascular consequences, including inhomogeneous ventricular repolarizing current, sinus node pacemaker current, and bundle-branch block. It also results in a variety of cardiac changes, including electrophysiological, structural, and contractile dysfunction and sudden cardiac death [21].

Cardiovascular autonomic dysfunction is mainly related to increased sympathetic activity via the right hemisphere of the insular cortex [31]. Alterations in autonomic nervous system function have been linked to ventricular and atrial arrhythmias. For example, both sympathetic-controlled HRV and parasympathetic-controlled HRV were decreased in patients with right hemisphere stroke involving reduced bilateral insula volumes [30, 32]. Further, these patients were more susceptible to cardiac complications such as arrhythmias and sudden death due to autonomic imbalance [33, 34].

The ECG changes observed following cerebral ischemia may be mediated through abnormal autonomic discharges caused by increased levels of brain natriuretic peptide, catecholamines, and NPY [21, 35, 36]. NPY released by sympathetic neurons can reduce vagal neurotransmission and directly influence ventricular myocyte excitability. NPY can also inhibit acetylcholine release during vagal nerve stimulation and bind to NPY receptors located on cholinergic ganglia and ventricular myocytes to inhibit *β*-receptor activity [37, 38]. NPY is widely distributed in both the central and peripheral nervous systems and is functionally related to regulation of blood pressure and circadian rhythms. Further, as a potent growth factor, NPY can cause cell proliferation in multiple systems, especially in the cardiovascular system [36], which is associated with cardiac hypertrophy via increased protein synthesis [39] and innervation-dependent changes in catecholamine-dependent chronotropic responsiveness [40]. Finally, there is increasing evidence that neurons can release NPY after myocardial infarction, which is involved in the progress of bilateral interventions targeting the sympathetic chain for arrhythmia modulation [24, 25, 41].

**Figure 1:**
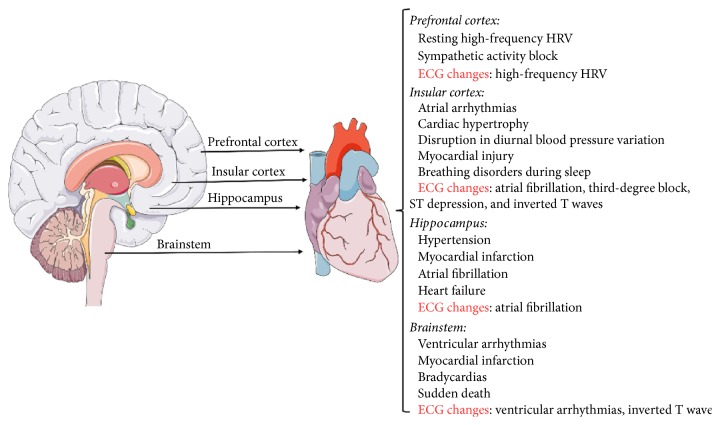
This figure shows that central and peripheral mechanism of the heart and brain interaction includes the clinical symptoms and ECG changes, but the effect of stellate ganglion or vagus and sympathetic nerves is not shown in this figure for an illustration. Of note, insular cortex, prefrontal cortex, hippocampus, and brainstem play an important role in interplay between the nervous system and the cardiovascular system.

The stellate ganglia play an important role in ventricular and atrial arrhythmia following insular cortex infarction. Structural changes in the stellate ganglion and cardiac nervous system occur as a result of chronic increases in sympathetic input and can cause arrhythmogenesis [42]. T wave changes are a key marker of ventricular tachyarrhythmia, myocardial infarction, and sudden cardiac death and are related to dispersion of repolarization during stellate ganglia activation [43, 44]. Circulating norepinephrine did not affect the ventricular dispersion of repolarization [42]. Further, atrial fibrillation, atrioventricular block, ectopic beats, sinus bradycardia, inverted T wave, and sudden cardiac death were more common in patients with a right insular lesion than in those with a left insular lesion. However, patients with bilateral acute insular lesions typically showed atrial fibrillation, third-degree block, ST depression, and inverted T waves [45]. Thus, it remains unclear whether the ECG changes associated with insular infarcts are related to the size of the insular lesion, as only partial insular infarcts or total insular infarcts have been taken into account [35] (Figure 1).

### 4.2. Cardiovascular Changes and the Brainstem

The brainstem (i.e., the external lateral parabrachial nucleus within the pons and rostral ventrolateral medulla) contributes to CNS processing of excitatory cardiovascular reflexes during cardiac sympathetic stimulation [46, 47]. The brainstem is an important regulator of cardiovascular responses to the interactive or exteroceptive environment, as well as vagal and sympathetic nerve activity [48]. Patients complicated with cardiac autonomic dysfunction due to brainstem lesions can exhibit ventricular arrhythmias, inverted T wave, myocardial infarction, bradycardias, and even sudden death [20, 24, 25, 48] (Figure 1).

### 4.3. Cardiovascular Changes and the Prefrontal Cortex

Resting high-frequency HRV (HF-HRV) is considered to reflect vagal blocking of sympathetic activity and can be used to assess the interaction of cardiovascular changes with the nervous system following cerebral injury after surgery. Interestingly, individual differences in resting HF-HRV can vary with resting state neural activity in the prefrontal cortex [49]. The prefrontal cortex region is of particular interest, as connectivity related to HF-HRV is shared by the default nervous network. However, increasing evidence suggests that HF-HRV is not directly related to the global resting state activity of intrinsic brain networks but rather to more localized connectivity. Thus, impaired autonomic cardiac control may occur following injury to brain regions such as the prefrontal cortex. For example, using a combination of electroencephalographic dynamics and instantaneous HR estimates to study emotional processing and cardiovascular autonomic responses in brain areas such as the prefrontal cortex and amygdala infract, Valenza et al. [50] found significant arousal-dependent changes between positive and negative stimuli, especially at intermediate arousing levels, throughout the prefrontal cortex (Figure 1).

### 4.4. Cardiovascular Changes and the Hippocampus

There is evidence that widespread hippocampal infarcts are associated with clinical symptoms of cognitive impairment or epilepsy. In a meta-analysis, Ishibashi et al. reported that the hippocampus was particularly vulnerable to ischemic insults under ultralow flow cardiopulmonary bypass [51]. Drabek et al. reported significant changes in the cortex, thalamus, hippocampus, and amygdala/piriform complex over time following early cerebral hyperperfusion and delayed cerebral hypoperfusion in rats [52]. Further, multiple mediators that accumulate during cerebral ischemia, as well as systemic factors such as acidosis, EAA, inflammatory factors, and ROS, have been implicated in both the no-flow and resuscitation periods [51, 53, 54].

A number of cardiovascular risk factors, including hypertension, myocardial infarction, atrial fibrillation, and heart failure, are also associated with large hemispheric brain infarcts [55]. Nevertheless, hippocampal infarcts play a prominent role, with postmortem evidence of large hemispheric brain infarcts being associated with heart failure and altered cardiovascular index [56, 57]. This is likely because large hemispheric infarcts, cardiovascular disease, and hippocampal infarcts have common risk factors [55], while the hippocampus and some other brain regions are particularly susceptible to cerebral ischemia and neuronal degeneration, resulting in the clinical syndrome of cognitive impairment, myocardial infarction, and heart failure [58] (Figure 1).

## 5. Summary

Improving our understanding of the mechanisms regulating postoperative brain injury and cardiovascular changes will aid in the development of appropriate therapeutic measures for cardiac surgery patients. Careful management of the operation period and favorable cardiopulmonary bypass conditions, as well as control of temperature, flow rate, pH, and hematocrit levels, can reduce the risk of cerebral infarction and thus avoid autonomic nerve damage and improve the prognosis of patients with cardiac surgery [59]. Similarly, furthering our understanding of the brain-heart axis and how brain lesions result in dynamic changes in ECG and HRV is important for facilitating the treatment of clinical symptoms in patients with brain injury after cardiac surgery.
